# Alcohol consumption and tobacco smoking associated with decreased antiretroviral therapy adherence among people living with HIV in Zambia: Evidence from a 2023 national NCDs/ HIV survey

**DOI:** 10.1371/journal.pone.0345368

**Published:** 2026-06-12

**Authors:** Cosmas Zyambo, Paul Somwe, Mwiche Musukuma, Chomba Mandyata, Phoebe Bwembya, Henry Phiri, Malizgani Chavula, Hikabasa Halwindi, Joseph Zulu, Wilbroad Mutale

**Affiliations:** 1 Department of Community and Family Medicine, School of Public Health, University of Zambia, Lusaka, Zambia; 2 Centre for Infectious Disease Research in Zambia, Lusaka, Zambia; 3 Department of Epidemiology and Biostatistics, School of Public Health, University of Zambia, Lusaka, Zambia; 4 Ministry of Health, Lusaka, Zambia; 5 Department of Health Policy, School of Public Health, University of Zambia, Lusaka, Zambia; Malaria Consortium, MOZAMBIQUE

## Abstract

**Background:**

In order to achieve the 95-95-95 goals and obtain optimal benefits from ART, PLWH must adhere to prescribed medication.

**Aim:**

Investigate factors associated with ART adherence in a clinical setting in Zambia.

**Methods:**

National cross-sectional study involving 193 clinics across all 10 provinces of Zambia. The primary outcomes assessed were ART adherence status, associated behavioral factors, and clinical characteristics. Logistic regression analysis was conducted to evaluate associations between these factors and ART adherence. Both unadjusted odds ratios (UOR) and adjusted odds ratios (AOR) were calculated, with adjustments made for relevant covariates, and all estimates reported with 95% confidence intervals.

**Results:**

Of the 5,204 PLWH, 7.1% were non-adherent to ART (9.7% males vs 5.9% females). Of those who were non adherent, 60% and 22% consumed alcohol and smoked tobacco respectively. In adjusted analysis, age; 45–59 (AOR: 0.5, 95% CI:0.32–0.79), 60+ (AOR: 0.26, 95% CI: 0.12–0.59) and education; Primary (AOR: 0.6, 95% CI: 0.4–0.9), secondary (AOR: 0.57, 95% CI: 0.4–0.82), college/university (AOR: 0.5, 95% CI: 0.29–0.89) were associated with decreased odds of being non-adherence to ART. In contrast, being male (AOR: 1.45, 95% CI:1.07–1.98), being in informal employment (AOR:1.65, 95% CI:1.15–2.36), having average yearly income; > $160 (AOR: 1.55, 95% CI: 1.16–2.06), Alcohol consumption; healthy consumption (AOR: 2.72, 95% CI: 1.9–3.89), unhealthy consumption (AOR: 2.76, 95% CI: 1.8–4.22), indeterminant consumption (AOR: 3.03, 95% CI: 2.04–4.5) and tobacco smoking (AOR: 95% CI: 2.03 1.42–2.9) were associated with increased odds of being non-adherence to ART.

**Conclusions:**

ART non-adherence among PLWH in Zambia is 7.1%, with higher rates in males. Substance use especially alcohol and tobacco are common among the non-adherent. The associations between alcohol consumption, tobacco smoking, and ART adherence highlight the potential value of targeted support strategies. These results can inform future longitudinal research and contribute to the development of evidence-based interventions

## Background

The benefits of antiretroviral therapy (ART) in prolonging life expectancy and improving clinical outcomes of People Living with HIV (PLWH) near to those without HIV are well established [1–3]; however, the increasing risk for the chronic co-morbidities, including cardiovascular diseases [4], depression [5,6], cancer [7] and other cardiometabolic abnormalities such diabetes and lipodystrophy [8,9] might negate the gains achieved so far. The optimal benefits of ART can only be attained when a PLWH is adhering to the prescribed medication [10,11]. Adherence refers to a patient’s ability to follow a treatment plan, take medications at prescribed times and frequencies, and adhere to dietary and medication restrictions [12]. Non-adherence to ART is associated with poor viral suppression, decreased CD4 + count, increased risk of antiretroviral drug resistance, and accelerated disease progression [13–15]. It is, therefore, critical to identify factors that lead to non-adherence and develop strategies to improve long-term adherence.

Hypertension, diabetes mellitus, stroke, and overweight/obesity significantly influence treatment adherence when analyzed within the framework of non-communicable diseases (NCDs) [16–18]. These conditions contributed to both positive and negative adherence patterns, with chronic illnesses like hypertension and diabetes linked to increased clinic visits and complex medication regimens which would increase the pill burden [18,19], stroke associated with cognitive or physical challenges [20,21], and overweight/obesity reflecting broader lifestyle and health engagement [22]. These findings underscore the need to factor in NCD-related variables for more accurate interpretation of adherence behaviors and the development of targeted interventions.

Alcohol consumption and tobacco use are more prevalent among PLWH than in the general population [23–26]. These have been seen to be a robust impediment to the treatment continuum, such as HIV testing uptake [27], retention in care [28,29], medication adherence [30–32], and viral suppression [26,32]. The mechanisms through which alcohol interrupts ART adherence are not yet known. However, intoxication impairs memory, organization skills, and other cognitive abilities, leading to missed doses [33,34]. It has been hypothesized that smoking interferes with the liver’s ability to process antiretroviral medications (ARVs), potentially reducing their effectiveness [35] and that smokers are more likely to experience side effects from ARVs, such as nausea and vomiting, which can lead to missed doses and poor adherence [35].

Studies on alcohol consumption and tobacco smoking in PLWH have been conducted [23,25,26,36–39]. Still, there are few studies on how alcohol consumption and tobacco smoking impact ART adherence in the treatment continuum not only in Zambia but in SSA [11,32,34,35,40], particularly in people with co-morbidities such as non-communicable diseases (NCDs). In Zambia, there is a scarcity of alcohol consumption [41–43] and tobacco smoking [44,45] studies in PLWH and no published data on alcohol consumption and tobacco smoking and its association with ART adherence in the PLWH with or without co-morbidities such as NCDs. This is a concerning data gap because HIV incidence and prevalence are high, and alcohol consumption and tobacco smoking are growing due to weak policies and a lack of interventions.

Globally, research has explored alcohol use among PLWH [46,47], but in SSA and Zambia, in particular there remains limited investigation into how any level of substance use, not just problematic use, impacts ART adherence. This gap is critical for PLWH with NCDs, where even minimal alcohol may exacerbate health risks and compromise treatment outcomes [46,47]. Recognizing this, our study emphasizes the importance of capturing any alcohol to identify early behavioral patterns that could precede more severe consequences.

In this study, we utilize the first-ever NCDs/HIV survey data to investigate factors associated with ART adherence in a clinical setting in Zambia. While the dataset focuses on NCDs among PLWH, our analytical emphasis on lifestyle risk factors such as alcohol and tobacco use reflect their impact on ART adherence, an outcome central to effective HIV treatment. The integration of NCD-related variables provides broader insight into the health complexities of PLWH and supports our aim of identifying potential targets for intervention. These findings may inform the design of targeted alcohol and tobacco interventions at the health facility level, applicable to PLWH both with and without NCDs.

### Methodology

#### Study design and setting.

**Fig 1:**
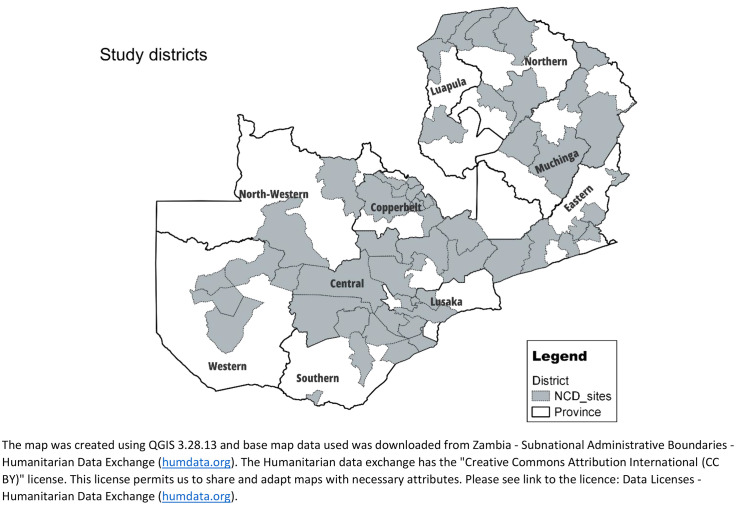
Provinces and districts of the study area in Zambia. The map was created using QGIS 3.28.13 and base map data used was downloaded from Zambia – Subnational Administrative Boundaries – Humanitarian Data Exchange (https:\\www.humdata.org). The Humanitarian data exchange has the “Creative Commons Attribution International (CC BY)” license. This license permits us to share and adapt maps with necessary attributes. Please see link to the licence: Data Licenses – Humanitarian Data Exchange (https:\\www.humdata.org).

This study was a nationally representative cross-sectional survey on non-communicable diseases (NCDs) among people living with HIV (PLWH) across the ten provinces of Zambia. Fig 1: shows the provinces and districts of the study area in zambia.

The survey included HIV-positive clients on antiretroviral therapy (ART) from health facilities providing ART services nationwide [48]. Eligible participants were HIV-positive adults (aged 18 years and above) on ART at the time of the survey and accessing ART services at the sampled health facilities using the national electronic health record system, SMARTCARE.

#### Sample size calculation and enrolment of participants.

To estimate the sample size, we used the prevalence formula, assuming an infinite population. With no reliable estimate of the prevalence of NCDs among PLWH, we assumed a prevalence of 50% to calculate the sample size. The estimated sample size was 385, with a two-sided 95% confidence interval and a 10% margin of error. To ensure adequate representation across different age groups (18–29, 30–44, 45–59, and 60 + years), rural-urban settings, and sex, we multiplied the calculated sample size by the total number of domains (i.e., 8), yielding a sample size of 3,080. To account for clustering and non-response, we multiplied the sample size by a design effect of 1.5 and adjusted for a 20% non-response rate, resulting in a total sample size of 5,775.

We employed a two-stage cluster sampling technique to select a nationally representative sample of health facilities providing ART services. A cluster was defined as a health facility (clinic) comprising HIV-positive patients. Our sampling frame included all health facilities providing ART services with a TXR of at least 500 from the electronic SMARTCARE system. In the first stage, facilities were stratified by province, and in the second stage, by rural-urban setting, creating a total of 20 strata. To allocate the total sample size of 5,775 across the 20 strata, with a fixed sample of 35 participants from each health facility, we used probability proportional to size to select 165 health facilities from the sampling frame. Participants missing outcome variables of interest were excluded from the analysis. Those who were missing outcome variables of interest were excluded from the analysis. Data from the participants was collected between 08/07/2023 to 18/07/2023. To reduce selection bias, four participants were systematically sampled per day, two in the morning and two in the afternoon. Because the main focus of the parent study was NCD’s among PLWH, all pregnant women and breastfeeding mothers were excluded from the study.

## Measures and variables

### Outcomes of interest

The primary outcome of interest was ART adherence: We used self-reporting to measure ART adherence as this has been shown to predict detectable viral load in a public ART program without routine plasma viral load monitoring in the SSA. We used the question in the questionnaire, “In the past 30 days, how many doses of ARVs has the participant missed?” and the responses were: 1) None, 2) one, 3) two, 4) three, and 5) four or more. Missing ≥ 1 dose over the 30 days preceding the survey was considered non-adherent. Consistent with prior research demonstrating that even a single missed dose can predict detectable viral load in public ART programs in SSA [49], we defined non-adherence as missing ≥1 dose in the 30-day period preceding the survey. This threshold was selected to enhance sensitivity in identifying individuals at risk of virologic failure, recognizing that strict adherence is critical for maintaining therapeutic drug levels and preventing resistance, especially in resource-limited settings [47]. The use of this conservative cutoff supports early intervention and aligns with global efforts to improve ART outcomes in high-burden regions [50]

### Covariates

**Socio-demographic** variables included sex (male or female), age, education level (No formal schooling, less than primary, Primary school completed, Junior Secondary school completed, Secondary Higher school completed, College/University completed, and postgraduate degree), income (<$160 vs ≥ $160); occupation (employed vs. unemployed), marital status (single, married or widowed/separated)

**Alcohol consumption** was assessed using the Alcohol Use Disorder Identification Test – C (AUDIT-C) based on the frequency of drinking, drinking category, and quantity of drinking. In the questionnaire, three questions were used to construct an AUDIT-C score.

a) **Frequency of drinking**: “During the past 12 months, how frequently have you had at least one standard alcoholic drink?” 1) Daily; 2) 5–6 days per week; 3) 3–4 days per week; 4) 1–2 days per week; 5) 1–3 days per month; 6) Less than once a month. A patient is assigned the following points: never (0), Less than monthly (1), Monthly (2), Weekly (3), Daily or almost daily (4).
b) **Quantity of drinking**: “During the past 30 days, when you drank alcohol, how many standard drinks on average did you have during one drinking occasion?” The patient stated the number of drinks. The patient is assigned the following points: None, I do not drink (0), 1 or 2 (0), 3 or 4 (1), 5 or 6 (2), 7–9 (3), or 10 or more (4).
c) **The drinking category**: “During the past 30 days, how many times did you have six or more standard drinks in a single drinking occasion?” The patient stated the number of drinks. A patient is assigned the following points: None, I do not drink (0), 1 or 2 (0), 3 or 4 (1), 5 or 6 (2), 7–9 (3), or 10 or more (4).

The AUDIT-C is scored on a scale of 0–12 (scores of 0 reflect none). Alcohol drinking is based on the AUDIT-C tool (AUDIT-C score ≤ 2 for women and ≤ 3 for men was classified as healthy alcohol consumption; AUDIT-C score ≥ 3 for women and ≥4 for men was classified as unhealthy alcohol consumption. Generally, the higher the AUDIT-C score, the more likely it is that the patient’s drinking is affecting his/her health and safety. Due to missing variables, we were unable to categorize some participants using the AUDIT score, even though they consumed alcohol, as a result, they were classified as indeterminate.

**Tobacco smoking:** Asked from the questionnaire “Do you currently smoke any tobacco products, such as cigarettes, Shisha, cigars or pipes?” (Yes/ No).

**Clinical variables** include a history of hypertension, history of metabolic diseases (diabetes and dyslipidemia), history of cardiovascular diseases (heart attack, stroke)

### Statistical analysis

Characteristics of ART adherence was calculated for the overall study participants. Continuous variables were reported as the means with the standard deviation (SD) or median with interquartile range (IQR), and categorical variables were reported as percentages. Using a logistic regression model, univariate and multivariable analyses were conducted to calculate unadjusted and adjusted odds ratios (ORs) and the corresponding 95% confidence intervals (CIs) for the association between the covariates and the outcome variable. The statistical significance level was p < 0.05 (two-tailed test). Analysis was performed using StataCorp. 2017. Stata Statistical Software: Release 15. College Station, TX: StataCorp LLC

### Ethical consideration

Ethics approval was obtained from UNZABREC (REF. 3007−2022). Subsequently, the protocol was submitted to the Zambia National Health Research Authority (Ref No: NHRA000011/12/09/2022) for registration and approval. Permission was sought from the Ministry of Health to allow their staff to participate in the study.

## Results

### Characteristics of the study participants

Of the 5,204 PLWH included in the analysis, 67.2% were females. More than 42% were between the age of 30–44 years, with almost 45% having attained secondary school education. More than half (56.3%) were married/cohabiting, 40% were in informal employment, and 46% were earning ≤ $160. Thirty-nine percent of the participants were consumers of alcohol (with 10.2% being identified as unhealthy drinkers, 17.8% as healthy drinkers, and 10.8% as indeterminant drinking). Almost a tenth (9.7%) of the participants smoked tobacco. In terms of co-morbidities, 18.3% had raised hypertension, 2.7% had diabetes mellitus, 10.4% had raised cholesterol, and 1.6% had experienced heart attack (Table 1).

**Table 1:** Characteristics of the study participants according to ART adherence status, national NCDs/ HIV survey 2023, Zambia.

Background characteristics	Overall	ART adherence status	
	N (% of total)	Adherent n (%)	Non-adherent n (%)
	5204 (100)	4833 (92.9)	371 (7.1)
**Gender**			
Female	3495 (67.2)	3290 (68.1)	205 (55.3)
Male	1709 (32.8)	1543 (31.9)	166 (44.7)
**Age (years)**			
Median (IQR)	43 (34-51)	43 (35-51)	39 (32-47)
18-29	724 (13.9)	663 (13.7)	61 (16.4)
30-44	2202 (42.3)	2024 (41.9)	178 (48.0)
45-59	1836 (35.3)	1727 (35.7)	109 (29.4)
60+	422 (8.1)	402 (8.3)	20 (5.4)
Missing	20 (0.4)	17 (0.4)	3 (0.8)
**Education**			
No formal schooling	1131 (21.7)	1025 (21.2)	106 (28.6)
Primary	1271 (24.4)	1193 (24.7)	78 (21.0)
Secondary	2331 (44.8)	2171 (44.9)	160 (43.1)
College/University	468 (9.0)	441 (9.1)	27 (7.3)
Missing	3 (0.1)	3 (0.1)	0 (0.0)
**Marital status**			
Never married	705 (13.5)	646 (13.4)	59 (15.9)
Currently married/Cohabitating	2929 (56.3)	2735 (56.6)	194 (52.3)
Divorced/Separated/Widowed	1551 (29.8)	1433 (29.7)	118 (31.8)
Missing	19 (0.4)	19 (0.4)	0 (0.0)
**Employment status**			
Formal employment	1041 (20.0)	985 (20.4)	56 (15.1)
Informal employment	2079 (40.0)	1901 (39.3)	178 (48.0)
Unemployed	1976 (38.0)	1845 (38.2)	131 (35.3)
Missing	108 (2.1)	102 (2.1)	6 (1.6)
**Average yearly income**			
≤4,000 ZMW	2389 (45.9)	2247 (46.5)	142 (38.3)
>4,000 ZMW	1210 (23.3)	1112 (23.0)	98 (26.4)
Missing	1605 (30.8)	1474 (30.5)	131 (35.3)
**Location**			
Rural	1366 (26.2)	1268 (26.2)	98 (26.4)
Urban	3838 (73.8)	3565 (73.8)	273 (73.6)
**Behavior variables**			
**Alcohol consumption**			
None	3183 (61.2)	3037 (62.8)	146 (39.4)
Healthy consumption	926 (17.8)	835 (17.3)	91 (24.5)
Un-healthy consumption	532 (10.2)	470 (9.7)	62 (16.7)
Indeterminant consumption	563 (10.8)	491 (10.2)	72 (19.4)
**Smoke tobacco**			
No	4701 (90.3)	4410 (91.2)	291 (78.4)
Yes	503 (9.7)	423 (8.8)	80 (21.6)
**Clinical variables**			
**BMI**			
Normal weight (18.5-24.9)	2915 (56.0)	2697 (55.8)	218 (58.8)
Underweight (≤18.4)	453 (8.7)	423 (8.8)	30 (8.1)
Overweight (25-29.9)	1166 (22.4)	1085 (22.4)	81 (21.8)
Obese (≥30)	590 (11.3)	550 (11.4)	40 (10.8)
Missing	80 (1.5)	78 (1.6)	2 (0.5)
Told by a doctor or other health worker that you have raised BP or HTN*			
No	3883 (81.7)	3592 (74.3)	291 (78.4)
Yes	872 (18.3)	818 (16.9)	54 (14.6)
Told by a doctor or other health worker that you have raised blood sugar or diabetes*			
No	1952 (93.5)	1837 (38.0)	115 (31.0)
Yes	136 (6.5)	131 (2.7)	5 (1.3)
Told by a doctor or other health worker that you have raised cholesterol*			
No	43 (89.6)	42 (0.9)	1 (0.3)
Yes	5 (10.4)	4 (0.1)	1 (0.3)
**Heart attack or stroke**			
No	5119 (98.4)	4754 (98.4)	365 (98.4)
Yes	85 (1.6)	79 (1.6)	6 (1.6)

*N for variables does not total 5204 due to prerequisite question response.

### ART adherence, Alcohol consumption and tobacco smoking

Among the 5,204 individuals surveyed, adherence to antiretroviral therapy (ART) was high, with 92.9% of participants. However, 7.1% were identified as non-adherent, with men showing a higher rate of non-adherence (9.7%) compared to women (5.9%). Non-adherent individuals tended to be younger, with a median age of 39 years. The age group most affected was 30–44 years, accounting for 48% of those who did not adhere to ART. Non-adherence was most prevalent among individuals with no formal education, accounting for 28.6% of cases. On employment status; individuals engaged in informal work (48%) and those who were unemployed (35.3%) represented the bulk of non-adherent cases. Income data was incomplete, with nearly one-third of participants opting not to disclose their earnings. However, among those who did report income, individuals earning less than 4,000 ZMW annually exhibited slightly higher rates of non-adherence. Alcohol use was common among non-adherent participants, reported by 60.6%, (24.5% identified as healthy drinkers, 16.7% as unhealthy drinkers, and 19.4% as indeterminate). Smoking prevalence among non-adherent PLWH was also high, with 21.6% reporting current tobacco use (Table 1).

### Factors associated with Non-adherence to ART

In univariate analysis, age, and educational status were significantly associated with decreased odds of non-adherence to ART. Males, employment, income, alcohol consumption, and tobacco smoking were significantly associated with increased odds of non-adherence to ART. In adjusted analysis, being aged between 30–44 years reduced the odds of being non-adherent (AOR: 0.78, 95% CI: 0.51–1.91); while being aged 45–59 years reduced the odds of being non-adherent to ART (AOR: 0.5, 95% CI: 0.32–0.79). The highest reduction in the odds of being non-adherent was estimated among participants aged above 60 years (AOR: 0.26, 95% CI: 0.12–0.59). With regards to education, having any level of education reduced the odds of being non-adherent to ART; Primary (AOR: 0.6, 95% CI: 0.4–0.9), secondary (AOR: 0.57, 95% CI: 0.4–0.82), college/university (AOR: 0.51, 95% CI: 0.29–0.89). Factors that increased the odds of being non-adherent included being male (AOR: 1.45, 95% CI; 1.07–1.98), not being in formal employment (AOR: 1.65, 95% CI; 1.15–2.36) and unemployment (AOR: 1.21, 95% CI: 0.78–1.85). Having an average yearly income of more than $160 compared to having an average yearly income of less than $160 increased the odds of being non-adherent to ART by 55% (AOR: 1.55, 95% CI: 1.16–2.06). Any level of alcohol consumption increased the odds of being non-adherent to ART. While healthy alcohol consumption increased the odds by 2.72 times (AOR: 2.72, 95% CI: 1.9–3.89), unhealthy consumption increased the odd by 2.76 times (AOR: 2.76, 95% CI: 1.8–4.22) and indeterminant consumption increase the odd by 3.03 times (AOR: 3.03, 95% CI: 2.04–4.5). Tobacco smokers (AOR: 2.03, 95% CI: 1.42–2.9) were associated with increased odds of being non-adherence to ART as compared to the non-smokers (Table 2).

**Table 2:** Factors associated with non- adherence to ART among PLWH, national NCDs/ HIV survey 2023, Zambia.

Background characteristics	Unadjusted (95% CI)	p-value	Adjusted OR (95% CI)	
**Gender**				
Female	1	**<0.001**	1	**<0.001**
Male	1.73 (1.39-2.14)		1.45 (1.07-1.98)	
**Age (years)**				
18-29	1	**0.005**	1	**<0.001**
30-44	0.96 (0.71-1.3)		0.78 (0.51-1.19)	
45-59	0.69 (0.5-0.95)		0.5 (0.32-0.79)	
60+	0.54 (0.32-0.91)		0.26 (0.12-0.59)	
**Education**				
No formal schooling	1	**0.009**	1	**0.009**
Primary	0.63 (0.47-0.86)		0.6 (0.4-0.9)	
Secondary	0.71 (0.55-0.92)		0.57 (0.4-0.82)	
College/University	0.59 (0.38-0.92)		0.51 (0.29-0.89)	
**Marital status**				
Never married	1	0.199		
Currently married/Cohabitating	0.78 (0.57-1.05)			
Divorced/Separated/Widowed	0.9 (0.65-1.25)			
**Employment status**				
Formal employment	1	**0.002**	1	**0.002**
Informal employment	1.65 (1.21-2.25)		1.65 (1.15-2.36)	
Unemployed	1.25 (0.9-1.72)		1.21 (0.78-1.85)	
**Average yearly income**				
≤4,000 ZMW	1	**0.016**	1	**0.01**
>4,000 ZMW	1.39 (1.07-1.82)		1.55 (1.16-2.06)	
**Location**				
Rural	1	0.33		
Urban	0.78 (0.62-0.98)			
**Behavior variables**				
**Alcohol consumption**				
None	1	**<0.001**	1	**<0.000**
Healthy consumption	2.27 (1.73-2.98)		2.72 (1.9-3.89)	
Un-healthy consumption	2.74 (2.01-3.75)		2.76 (1.8-4.22)	
Indeterminant consumption	3.05 (2.26-4.11)		3.03 (2.04-4.5)	
**Smoke tobacco**				
No	1	**<0.001**	1	**<0.000**
Yes	2.87 (2.19-3.74)		2.03 (1.42-2.9)	
**Clinical variables**				
**BMI**				
Normal weight (18.5–24.9)	1	0.840		
Underweight (≤18.4)	0.88 (0.59-1.3)			
Overweight (25–29.9)	0.92 (0.71-1.2)			
Obese (≥30)	0.9 (0.63-1.28)			
**Told by a doctor or other health worker that you have raised BP or HTN**				
No	1	0.173		
Yes	0.81 (0.6-1.1)			
**Told by a doctor or other health worker that you have raised blood sugar or diabetes**				
No	1	0.254		
Yes	0.61 (0.24-1.52)			
**Heart attack or stroke**				
No	1	0.980		
Yes	0.99 (0.43-2.28)			

## Discussion

In the national NCDs/HIV health facility survey conducted in Zambia, we observed significant levels of non-adherence to ART among PLWH. Beyond documenting the general prevalence of alcohol consumption and tobacco use, we also identified these behaviors as disproportionately higher among non-adherent individuals. This suggests a critical interplay between substance use and treatment adherence, yet existing literature largely overlooks this vulnerable subpopulation. Given the implications for treatment outcomes and public health strategy, there is a compelling need to further investigate alcohol consumption among PLWH, particularly its influence on ART adherence. We also showed factors associated with non-adherence to ART. The study adds four key findings to the body of knowledge on adherence and alcohol consumption and tobacco use. Firstly, 7.1% of the PLWH were non-adherent to ART in Zambia. Secondly, the prevalence of alcohol consumption is 38.8% (with 10.2% being identified as unhealthy drinkers, 17.8% healthy drinkers, and 10.8% indeterminant drinking) and tobacco smoking is 9.7% consumed alcohol. Thirdly, of those who were non-adherence, more than half (60%) reported consuming alcohol, with 16.7% being classified as unhealthy drinkers, 24.5% as healthy drinkers, and 19.4% as indeterminate drinkers, and almost 22% reported to be smokers. Fourthly, males who were unemployed or in informal employment, earning an average yearly income of>$160, consumed alcohol, and smoked tobacco were more likely to be non-adherent to ART. It’s worth noting that, any classification of consumption of alcohol (unhealthy, healthy, and indeterminate) was associated with non-adherence to ART.

The study has shown that 7.1% of PWLH were non-adherent to ART. PLWH need ≥ 95% ART adherence to achieve viral suppression [51]. The prevalence is lower than what is obtained in other parts of Zambia, nyimba and Monze district (16.5%) [48,52], kasama district (18%) [53] and chikankata district (58.4%) [54]. Unexpectedly, our study has shown much lower non-ART adherence than other studies in Zambia because there have been strategies that have implemented to enhance adherence over time. It’s possible that the non-adherence prevalence has reduced from the time these studies were done. Our result compared to other countries, shows that adherence is much lower than that obtained in other countries such Egypt, [55] Nigeria [56] and other sub-Saharan countries [57]. Although this study demonstrates much-improved adherence compared to other studies conducted in Zambia and other sub-Saharan countries, it remains necessary to identify the key predictors of non-adherence to ART in order to implement targeted interventions.

In our study, men were more likely to be non-adherent to ART; this is in conformity to other studies that reported similar results [58,59]; literature reviews have suggested otherwise [60]. The plausible reason men are non-adherent might be that men are less likely to access healthcare facilities than women due to poor health-seeking behavior influenced by gender norms and stigma. Increased age was associated with adherence. Those who were older than 18–24 were more likely to be adherent to medication; this is consistent with findings from other studies [59,61]. It has been argued that the younger PLWH experience discrimination in healthcare settings, which may result in disengagements [62]]. Young people often face mental health issues like depression and anxiety, which can hinder their adherence to treatment regimens [63]. Additionally, the side effects of ART can be particularly challenging for them to manage, leading to non-adherence [63].

Furthermore, the study found that adults living with HIV who had an educational level of primary school, secondary, and college/university were more likely to adhere to ART compared to those with no form of education. This finding aligns with other studies [64–66] conducted Nigeria, Togo and Nepal. It is possible that a low level of education among individuals can lead to a lack of awareness about HIV and its treatment, which may result in higher non-adherence to ART. Individuals who were unemployed or informal were more likely to be non-adherent than those who were formal employment. This conforms with a plethora of studies showing that employment status significantly impacts adherence to ART [67–69]. The argument is that employment provides financial stability, enabling individuals to afford transportation to healthcare facilities and purchase necessary medications. In addition, workplaces offer support systems, such as health insurance and employee assistance programs, that can significantly facilitate adherence to ART [70]. With regards to income, our study found an inverse relationship between a participant average yearly income and adherence to ART. Our study showed that those with an average yearly income of greater than $160 were more likely to be non–adherent than those with an average yearly income of less than $160. This could be due to underreporting or overreporting of income earned. Income variables in Zambia and Africa, in general, are not good indicators to use as they are not accurate reflections of the income that the person gets. Even those in employment have other external income. It is suggested that the wealth index is the best measure of income. Unfortunately, we were unable to collect that data.

Our study showed that 38.8% of PLWH on ART are current consumers of alcohol and the prevalence is almost twice that of the general population of Zambian adults [71]. This prevalence is higher than what is obtained in another study in Zambia (36.8%) [41], Tanzania (29.1%) [72], and Uganda (33%) [32] but lower than in the New Orleans alcohol study (58.9%−73.7%) [73]. A tenth of our study participants consumed an unhealthy amount of alcohol in the 30 days leading to the survey. However, this estimate was lower than what other similar studies in Zambia found [39,41], but higher than what’s reported in the neighboring countries such as Malawi and Zimbabwe [39,74]. Consistent with existing literature on alcohol use and ART adherence, our findings indicated that more than 60% of those who were non-adherent to ART reported current alcohol use, and 16.7% screened positive for unhealthy drinkinge.

Even after adjusting for socio-demographic, behavioral, and clinical factors, alcohol drinking remained a strong predictor for non-adherence to ART in PLWH. This finding is consistent with several studies elsewhere [58–60]. This observation is plausibly explained by the effects of alcohol consumption on cognitive function, which can impair individuals’ ability to remember to take their medication on time [31,33]. It also leads to risky behaviors and poor decision-making, which can interfere with adherence to ART [31,33]. Alcohol use is often linked to mental health issues such as depression and anxiety, which can further impact adherence [6]. It is important to recognize that in Zambia, many households brew cheap illicit alcohol to earn a living, and access to these alcoholic beverages is unrestricted, providing one with easily accessible and affordable alcohol to buy [75]. Without strengthening and reinforcing the alcohol policies to regulate alcohol consumption in these places, many PLWHs will continue to have access to these cheap alcohols leading to non-adherence of ART.

Our study found that 9.7% of the PLWH smoke tobacco; notably, this prevalence is lower than what is recorded in other studies of the same settings [26,76,77]. Previous studies have shown that PLWH smokes tobacco 2–3 times more than the general population [44,78,79]; under-reporting might explain the lower prevalence. In the context of numerous studies that have demonstrated an association between ART adherence and tobacco smoking [80–82], It is consistent with prior findings that 22% of PLWH who were non-adherent to ART in our study were current smokers. While additional research is needed to identify the mechanisms, nicotine dependence may be associated with a generalized disregard for the health effects of behavior change, including ART adherence. It is well documented that individuals with nicotine dependence are less likely to achieve smoking cessation [83]. Although our study did not conduct the Fagerstrom score to measure the nicotine dependence, the individual dependance to nicotine is a strong predictor of the failure to achieve stable changes in smoking behavior. Regardless of the underlying mechanisms, our study provides an opportunity to identify PLWH who are at risk for poor ART adherence and would benefit from early interventions aimed at maintaining adherence through smoking cessation.

This study is without limitations. Due to the observational design, we were unable to assess changes in these factors over time. This design allowed us to identify factors associated with ART non adherence but did not permit us to establish causality. Although we adjusted for known confounders in the multivariable model, the potential for residual confounders inherent in observational studies remains, which might affect the interpretation of our findings. Alcohol consumption, tobacco use and ART adherence were self-reported, relying on the honesty and trustworthiness of respondents. In studies relying on self-reported data especially ART adherence data, several cognitive and social factors can compromise reliability. Recall bias is a frequent issue, as participants may struggle to accurately remember their medication-taking behaviors [84].This often leads to underreporting or overreporting of ART adherence. Social desirability bias further complicates matters as respondents may consciously or unconsciously exaggerate their compliance in an effort to present themselves as responsible or cooperative, especially in clinical settings like ours where adherence is closely monitored [84,85]. Additionally, inconsistencies in how individuals interpret survey questions can lead to varied responses based not on actual behavior, but on subjective understanding [86]. These limitations highlight the need for complementary objective measures to validate self-reported data through either electronic monitoring devies, direct observation therapy or biological biomakers. While this study emphasizes behavioral factors such as non-adherence, prior research suggests that alcohol may independently enhance HIV replication. However, a key limitation of our analysis is the lack of viral load data, which restricts our ability to directly examine alcohol’s biological effects on HIV progression. Furthermore, the study was conducted in Zambia, so the findings may not be generalizable to other countries. Despite these limitations, this is the first Zambia nationally representative data to underscore non-adherence to ART among PLWH and address gaps in the literature regarding the impact of alcohol consumption and tobacco smoking on ART adherence.

A key methodological limitation of this study lies in the stringent definition of ART non-adherence, which we defined as missing one or more doses in the past 30 days. While this threshold may overestimate non-adherence compared to more lenient definitions (e.g., < 95% adherence or missing ≥2 doses), it was selected based on evidence that achieving and maintaining viral suppression requires adherence levels of at least 95%. Missing even a single dose equivalent to ~3.3% non-adherence can compromise treatment effectiveness, particularly with first-line regimens commonly prescribed in sub-Saharan Africa. Our approach aligns with studies showing that even occasional missed doses may lead to virological rebound or resistance, especially when compounded over time. Nonetheless, we recognize that newer ART regimens may offer greater pharmacologic forgiveness

In summary, our analysis highlights a concerning level of non-adherence to ART among PLWH in Zambia, with a higher prevalence observed in males. Substance use particularly alcohol consumption and tobacco smoking was notably prevalent among the non-adherent participants, suggesting a powerful linkage between lifestyle factors and treatment outcomes. Adjusted analyses further emphasizes that socio-demographic characteristics such as younger age, lower education levels, informal employment, and higher income are associated with increased odds of non-adherence. In contrast, older age and higher educational attainment were protective. These results underscore the urgency of targeted interventions that address substance use and social determinants in ART adherence strategies, with a particular focus on vulnerable subgroups. Strengthening behavioral health integration into HIV care could be instrumental in improving treatment retention and overall health outcomes.
